# Rice Quality: A Multidimensional Evaluation Integrating Ecology, Management and Genetic Regulation

**DOI:** 10.3390/foods15050813

**Published:** 2026-02-26

**Authors:** Wengong Huang, Dongmei Shi, Aihua Cheng, Guofeng Chen, Feng Liu, Jiannan Dong, Jing Lan, Wei Guo, Baohai Liu, Chuanying Ren

**Affiliations:** 1 Safety and Quality Institute of Agricultural Products, Heilongjiang Academy of Agricultural Sciences, Harbin 150086, China; huangwengong1736@163.com (W.H.); shidongmei@126.com (D.S.); aihcheng@163.com (A.C.); hljjiance@163.com (G.C.); 13766881790@126.com (F.L.); jiannan_dong@163.com (J.D.); lanjing1968@163.com (J.L.); guoweixinwei@126.com (W.G.); shslbh@126.com (B.L.); 2 Key Laboratory of Quality and Safety of Cereals and Their Products, State Administration for Market Regulation, Harbin 150086, China; 3 Food Processing Research Institute, Heilongjiang Academy of Agricultural Sciences, Harbin 150086, China

**Keywords:** rice quality, expression form, influencing factors, associated genes, biological breeding improvement

## Abstract

With global economic development and rising living standards, expectations regarding the quality of staple rice have become increasingly multifaceted. This shift has imposed more stringent demands on high-quality rice breeding and field management and has stimulated research into the mechanisms underlying changes in rice quality. This article explores how assessments of rice quality have evolved from a primary emphasis on appearance, eating and processing quality to include stronger requirements for nutritional value and safety. In rice production systems, quality outcomes are influenced by interactions among genetic traits, ecological factors and field management practices. Through genetic improvement, biological breeding techniques and precise field management, improvements in appearance, eating and nutritional qualities can be achieved. Although climate change is an uncontrollable external factor affecting rice quality, constructing multi-factor dynamic simulation models that target key genes has been proposed as a strategy to enhance stress resistance and guide rice breeding. Rice safety and quality depend on the rational use of pesticides in terms of timing and dosage, which can help mitigate disease and insect resistance while reducing the risks associated with pesticide residues and toxins. Furthermore, the application of artificial intelligence technologies in biological breeding and field management can shorten breeding cycles, improve disease and pest outbreak prediction and support the timely formulation of treatment prescriptions.

## 1. Introduction

The major rice-producing regions of the world are concentrated in Asia, which accounts for approximately 90 of the global rice cultivation area. Rice production is primarily distributed across India, China, Indonesia, Bangladesh, Thailand, Vietnam and other Asian countries. Approximately 30% of the global rice-sown area is located in India, ranking it first worldwide, while its contribution to global rice production is estimated at 21.7%, placing it second globally [1]. Although China accounts for approximately 23% of the world’s rice planting area, it ranks first in total rice production. This higher productivity has been attributed to the widespread adoption of high-performing rice varieties, advanced cultivation techniques and favourable growing environments. Rice is the staple food for approximately half of the world’s population and represents a primary source of carbohydrates. Therefore, rice quality is paramount [2].

Rice quality is widely used as an indicator of rice production value, and its formation is influenced by both varietal genetic traits and the cultivation environment. From a genetic perspective, substantial differences have been observed among rice varieties in protein content, amylose content and chalkiness traits. From an environmental perspective, ecological factors such as temperature, light availability and water conditions directly influence the development of rice quality attributes. Additionally, soil fertility, the level of field management and the intensity of disease and pest occurrence have been shown to impact rice quality outcomes. The most crucial factor is climate, temperature affects starch synthesis, light is related to the accumulation of photosynthetic products, and water is directly related to grain plumpness. Next is the soil characteristics, pH value, organic matter, and trace elements will all penetrate into the components of rice. The management of growth environment is also important, such as the management of water layers. Special attention needs to be paid to the correlation between various factors, such as the possibility of increased temperature exacerbating the effects of drought, and soil pH affecting the accumulation of trace elements. Perceptions and evaluation criteria for rice quality also vary across countries, regions and urban contexts. In Southeast Asia, high-quality rice is commonly characterised by nutritional value, softness and aroma, whereas in South Asia, high-quality rice is primarily defined by appearance attributes (including evenness, whiteness, long grain), satiety and aroma [3].

The evaluation of rice eating quality is primarily conducted using sensory evaluation methods [4,5], instrumental analytical approaches (including assessment of aroma [6], taste [7], nutritional components [8]) and physicochemical index-based methods. In China, combined approaches integrating ‘artificial sensory evaluation’ and ‘machine simulated chewing’ systems are being explored to quantitatively assess rice eating quality. According to the national standards for grain and oil quality in China, rice is classified into japonica rice, indica rice and glutinous rice based on differences in grain morphology and quality characteristics. The Yangtze–Huaihe River region and Northeast China are recognised as major japonica rice-producing areas, while Hunan and Guangdong are recognised as major indica rice-producing regions [9]. Although some researchers reviewed traditional rice quality traits and [4] examined rice quality from a consumer perspective, recent reviews integrating evaluation methods with environmental modulation and breeding strategies under climate change conditions remain limited [3]. Therefore, this review is intended to (1) synthesise modern rice quality evaluation frameworks, (2) analyse genetic × environment × management (G × E × M) interactions influencing rice quality and (3) provide evidence-based strategies for rice quality improvement.

## 2. Methodology

This review article comprehensively examines rice quality evaluation methods, ecological and management factors influencing quality, rice quality-related genes and the enhancement of rice quality through biological breeding, with particular attention to their inter-relationships. The literature was systematically retrieved from Web of Science, PubMed, X-MOL, MDPI and SpringerLink using the keywords ‘rice quality’, ‘influencing factors’, ‘quality-related genes’ and ‘biological breeding improvement’, with a primary focus on publications from 2020 to 2024. The titles and abstracts were independently screened by two reviewers. Duplicate records and non-English literature were excluded. The selected studies were then classified according to predetermined thematic categories. Subsequently, the full texts were thoroughly read, summarised, synthesised and analysed in detail. Of the 232 articles initially identified, 93 met the inclusion criteria and were selected for a detailed review. These selected studies provide a solid foundation for the analyses and discussions presented in this article.

## 3. Evaluation of Rice Quality

**Figure 1:**
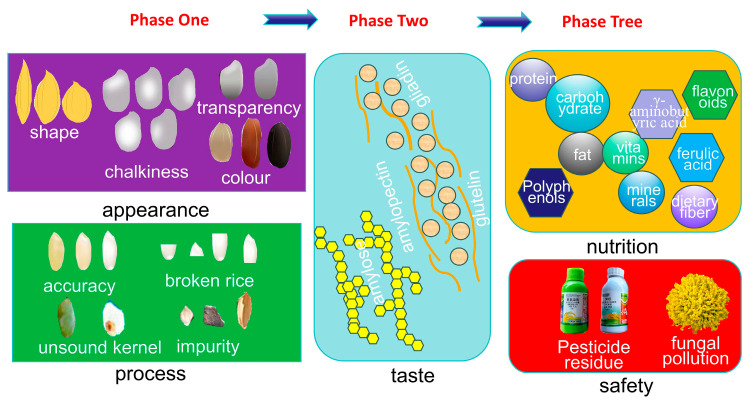
Indicators for the evaluation of rice quality.

Rice quality evaluation has become increasingly comprehensive at both national and international levels, encompassing appearance quality, eating quality, processing quality, nutritional quality and safety quality (Figure 1). Between 1980 and 1990, rice quality evaluation was in its initial stage, with consumer evaluations primarily focused on appearance attributes. From 1990 to 2000, assessments entered a second stage, in which sensory descriptors, such as ‘delicious’ and ‘tasty’, were commonly used to define high-quality rice. Since 2000, rice quality evaluation has progressed to a third stage, with an increasing emphasis on nutritional value and food safety. With rising living standards, consumer expectations of rice quality have correspondingly increased. Enhancing the eating quality of rice has become an important measure to meet consumer demand for improved dietary satisfaction and overall quality of life [10].

### 3.1. Appearance Quality of Rice

Rice appearance is a complex and multifaceted trait. Appearance quality represents a key determinant of overall rice quality, reflecting its capacity to attract consumers. Principal attributes include grain shape, chalkiness, transparency and colour [11].

Typically, japonica rice exhibits a short and round grain morphology, whereas indica rice is characterised by slender grains. Glutinous rice may be derived from japonica or indica. According to international standards, a length-to-width ratio > 3.0 defines a long-grain shape. In China, high-quality indica rice is required to have a length-to-width ratio ≥ 3.0. The national standard GB/T 17891-2017 for ‘high-quality rice’ classifies high-quality indica rice into three categories based on the length of brown rice: long grain (length > 6.5 mm), medium grain (length 5.6–6.5 mm) and short grain (length < 5.6 mm).

Chalkiness of rice refers to opaque regions within the rice endosperm and can be categorised as abdominal chalkiness, heart chalkiness, back chalkiness and other forms, with abdominal chalkiness being the most common. Chalkiness is negatively correlated with transparency, a trait generally preferred by consumers. The reduction in chalkiness constitutes a primary objective in rice quality improvement. Chalkiness formation involves complex molecular and physiological mechanisms, primarily controlled by genetic and environmental conditions. At the structural level, chalkiness arises from a loose arrangement of starch and protein in the endosperm, resulting in an abnormal endosperm structure [12]. Starch granules within chalky regions are irregular in size, loosely packed with significant intergranular gaps and exhibit oval or round shapes [13].

Rice transparency refers to the capacity of rice grains to transmit light, which is directly associated with both appearance and eating quality. Rice grains with high transparency typically exhibit reduced chalkiness and uniform morphology. Cooked rice derived from such grains shows lustre and taste, characteristics generally preferred by consumers. Transparency is minimally affected in grains with a thickness ≥1.9 mm, while thinner grains show a significant reduction in transparency. A lower degree of chalkiness is associated with higher transparency, while excessively high seeding densities have been shown to increase starch content and reduce transparency [14]. Negative correlations have been observed between rice grain transparency and grain moisture, amylose content and the presence of cavities within starch granules. As moisture decreases, cavities within starch granules become more transparent [15].

In conventional rice, the caryopsis seed coat after shelling is light yellowish-brown. Coloured rice, conversely, exhibits caryopsis in shades of black, purple, red and other hues. Brown rice from coloured varieties is enriched in naturally occurring water-soluble pigments, minerals and other nutrients [16]. These varieties also contain higher levels of polyphenols and flavonoids, which have been associated with antioxidant activity, lipid regulation and blood sugar control [17]. The composition of anthocyanins in the rice cortex varies substantially between different coloured varieties, while minor differences are observed among rice of the same colour, reflecting the influence of varietal genetics, growth environment and planting conditions [18].

### 3.2. Eating Quality of Rice

The eating quality of rice refers to the integrated characteristics of rice in terms of taste, aroma and appearance following appropriate processing and cooking. It constitutes a key parameter for assessing the overall quality of rice as a food product. The eating quality of rice is affected by multiple factors, including appearance, taste, aroma, hardness, viscosity, elasticity, chewiness and smoothness [19]. These attributes are both subjective and measurable. Eating quality represents a central focus of rice quality research, breeding and cultivation management, as it directly affects the taste, appearance and overall consumption experience. High-quality rice is typically characterised by sensory attributes described as ‘soft, elastic, fragrant and smooth’. In China, a comprehensive rice quality evaluation system has been established, supported by national standards such as GB 1350-2009 (‘paddy’) [20], GB/T 17891-2017 (‘high-quality paddy’) [21] and GB/T 1354-2018 (‘rice’) [22], which standardise evaluation procedures and criteria for rice quality. Other countries, including Japan and Thailand, have also developed their own rice quality evaluation systems. For example, in Japan, the eating quality of japonica rice varieties, such as Yueguang, is rigorously assessed using both sensory evaluation and physicochemical index measurements.

Amylose and protein content are recognised as key determinants of rice quality, with both parameters generally showing negative correlations with sensory quality attributes [20]. Rice aroma is composed of more than 300 volatile compounds, among which 2-acetyl-1-pyrroline, aldehydes, heterocyclic compounds and alcohols play major roles in aroma formation [21,22]. Under typical conditions, the amylose content of rice ranges from approximately 14–20%, and lower amylose levels are commonly associated with improved eating quality. When the amylose content exceeds 20%, interactions between starch and lipids, as well as starch and proteins, are intensified, thereby affecting starch granule expansion and water absorption. These interactions also influence gloss, viscosity and hardness, leading to a significant reduction in eating quality [23]. Rice proteins are mainly classified into albumins (water-soluble), globulins (salt-soluble), glutelins (alkali-soluble) and glutenin (alcohol-soluble) [24], with glutelins accounting for more than 80% of the total protein content. The three-dimensional network structure formed by glutelins surrounds starch granules, thereby restricting water penetration and starch swelling [25]. Therefore, a lower glutelin-to-gliadin ratio is associated with improved gel consistency, enhanced pasting properties and better eating quality [26]. Additionally, cooking has been shown to promote protein aggregation in rice, resulting in a more compact structure and a significant increase in protein particle size (raw rice: 304.33 nm; cooked rice: 565.17 nm). This structural alteration is accompanied by a substantial reduction in protein solubility and foaming capacity, thereby affecting rice eating quality [27,28].

Different cooking methods have been shown to significantly influence the eating quality of rice, such as conventional cooking and high-pressure steam cooking. Conventional rice cookers are generally suitable for rice. However, for whole-grain brown rice and black rice, in which the seed coat is intact, traditional cooking methods are often insufficient to facilitate adequate water penetration. Consequently, starch within the grain does not fully absorb water or undergo gelatinisation, leading to poor taste [29]. Conversely, high-pressure steam cooking has been shown to effectively disrupt the barrier imposed by the seed coat, thereby enhancing water penetration. This method increases starch solubility and swelling capacity, reduces the starch gelatinisation temperature and induces a transformation of the starch crystalline structure from type A to type V, ultimately resulting in a softer texture and improved eating quality [30,31].

Pretreatment of rice has been shown to improve its eating quality. In rice samples subjected to superheated steam treatment, amylose and protein contents were significantly reduced, the average starch particle size increased and the starch structure became more compact and ordered. These structural changes resulted in marked improvements in gel hardness, viscoelasticity and strength, leading to improved eating quality [32]. Similarly, prebaking treatment was found to increase the levels of damaged starch and resistant starch, improve water absorption and shorten optimal cooking time. This treatment also reduced rice hardness and adhesiveness, yielding a softer texture in cooked grains [33].

### 3.3. Rice Processing Quality

The rice processing quality is primarily determined by processing accuracy, broken rice rate, imperfect grain content, moisture content and impurity content, all of which are closely associated with eating quality [34]. Processing accuracy of rice is classified into three categories: fine milling, moderate milling and substandard milling [35]. The affinity of the rice grain cortex, embryo and endosperm to eosin Y–methylene blue staining differs, showing blue-green, purple and red colouration, respectively [36]. The broken rice rate quantifies the proportion of fractured grains and affects both the visual quality and market value of rice. Imperfect grains, comprising immature and diseased grains, negatively affect appearance, nutritional content and edible quality [37]. In China, the maximum allowable moisture content is 14.5 for indica rice and indica glutinous rice and 15.5% for japonica rice and japonica glutinous rice. Impurities include rice straw, barnyard grass, soil, sand, stones and other foreign matter. According to national standards, the total impurity content in rice must not exceed 0.25%, with inorganic impurities limited to ≤0.02%. The first-class quality indicators for Chinese rice are summarised in Table 1.

Factors affecting rice processing quality include variety, cultivation practices and processing technology. Different rice varieties possess distinct genetic characteristics, including protein content, amylose content and gel consistency, which collectively affect eating-quality traits such as taste, viscosity and hardness. Chalkiness has been observed to increase with higher protein content and decrease with higher starch content [38]. Cultivation practices also play a critical role. Appropriate planting density and scientifically managed fertilisation have been shown to improve grain quality, while pest and disease incidence negatively affect grain plumpness. Processing technology is another key determinant. Temperature and pressure control during milling and polishing significantly influenced the retention of nutrients within the rice endosperm. In the production of first-class rice, precise control of these parameters is employed to maximise nutrient preservation. Finally, storage conditions are critical for maintaining rice quality. Adequate ventilation and appropriate temperature help preserve the quality of rice and prevent spoilage from mildew or insect infestation.

### 3.4. Nutritional Quality of Rice

**Figure 2:**
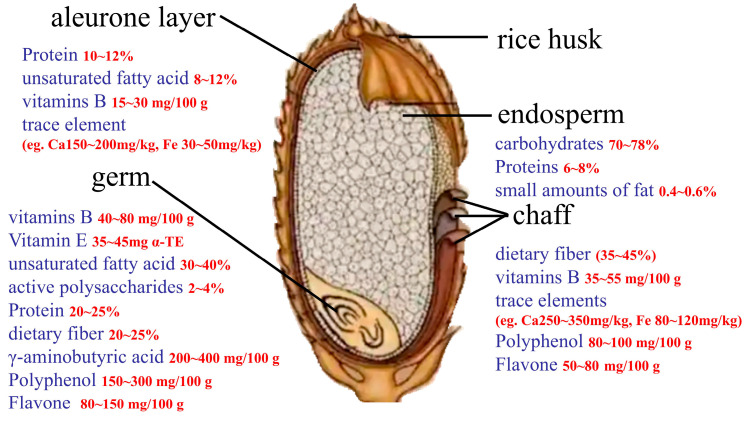
Nutrient composition across different rice structures.

Nutritional quality in rice is primarily determined by its content of macronutrients, such as carbohydrates, fats, dietary fibre, protein, vitamins and minerals [39]. Additionally, the bran and embryo are rich in bioactive compounds, including essential amino acids, phenolic compounds, flavonoids, vitamin E, γ-oryzanol and γ-aminobutyric acid (GABA). These compounds contribute to antioxidant capacity, enhancing storage stability and may provide beneficial effects on human health [40,41]. These nutrients and bioactive compounds are differentially distributed across rice structures (Figure 2), and their concentrations vary significantly among these structures. Protein in rice is predominantly composed of glutelins, with content ranging from 6 to 8% in the endosperm and 8–12% in the aleurone layer. In the germ, the protein content is considerably higher, reaching 20–25%. Fat content is low in the endosperm (0.4–0.6%), while the aleurone layer contains 8–12% fat, predominantly unsaturated fatty acids, and the germ is particularly rich in unsaturated fatty acids, with content ranging from 30 to 40 elements, particularly calcium and iron, are primarily located in the chaff and aleurone layer. Calcium content in the chaff was measured at 250–350 mg/kg, and iron content at 80–120 mg/kg. The bioactive compound GABA was concentrated in the germ, with levels reaching 200–400 mg/100 g.

In aleurone layer, protein 10~12% (*n* = 12 studies, 56 cultivars); unsaturated fatty acid 8~12% (n = 8 studies, 16 cultivars); vitamins B 15~30 mg/100 g (n = 6 studies, 13 cultivars); Ca 150~200 mg/kg and Fe 30~50 mg/kg (n = 6 studies, 9 cultivars); in germ, vitamins B 40~80 mg/100 g (n = 4 studies, 6 cultivars), vitamin E 35~45 mg α-TE (n = 5 studies, 10 cultivars), unsaturated fatty acid 30~40% (n = 11 studies, 18 cultivars), active polysaccharides 2~4% (n = 3 studies, 6 cultivars), protein 20~25% (n = 15 studies, 28 cultivars), dietary fibre 20~25% (n = 14 studies, 24 cultivars), γ-aminobutyric acid 200~400 mg/100 g (n = 6 studies, 10 cultivars), polyphenol 150~300 mg/100 g (n = 8 studies, 16 cultivars), flavone 80~150 mg/100 g (n = 6 studies, 22 cultivars); In chaff, dietary fibre 35~45% (n = 6 studies, 10 cultivars), vitamins B 35~55 mg/100 g (n = 8 studies, 12 cultivars), Ca 250~350 mg/kg and Fe 80~120 mg/kg (n = 5 studies, 10 cultivars), polyphenol 80~100 mg/100 g (n = 8 studies, 14 cultivars), flavone 50~80 mg/100 g (n = 7 studies, 14 cultivars); In endosperm, carbohydrates 70~78% (n = 7 studies, 10 cultivars), proteins 6~8% (n = 5 studies, 8 cultivars), fat 0.4~0.6% (n = 10 studies, 16 cultivars).

**Figure 3:**
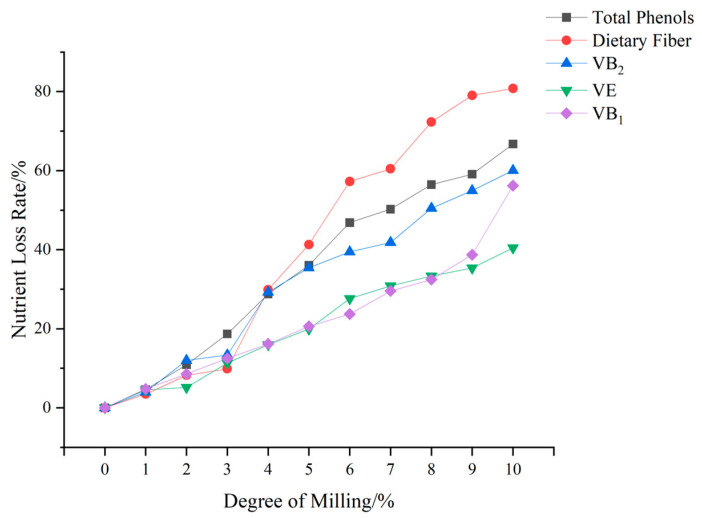
Nutrient loss rates in brown rice at different degrees of milling.

Factors influencing the nutritional quality of rice include processing accuracy and variety. Brown rice, in which embryos and cortexes are retained, contains higher levels of protein, vitamins, minerals and bioactive compounds. Although polished rice exhibits superior taste, substantial nutrient losses occur during processing. Specifically, when the milling degree of brown rice increases from 5% to 9%, significant reductions in protein, dietary fibre and mineral content are observed, while carbohydrate content increases. Consequently, rice with 5% milling and embryo retention is recommended for consumption [42]. This form of rice, known as reserved germ rice, is produced by moderately processing brown rice to remove the cortex while retaining the germ, thereby combining enhanced nutritional value with favourable taste characteristics (Figure 3) [43]. Furthermore, speciality rice varieties, such as black rice and red rice, are rich in anthocyanins, cyanidins, paeoniflorin, proanthocyanidins and other antioxidant compounds, which are predominantly localised in the cortex [44,45].

### 3.5. Safety Quality of Rice

The safety of rice is primarily determined by two factors: chemical contamination and mycotoxin contamination. Chemical contamination includes pesticide residues and heavy metals. Throughout the rice growth cycle, various pesticides are applied to prevent and control diseases, pests and weeds, as well as to regulate growth (Table 2). For the detection and mitigation of pesticide residues, high-performance liquid chromatography with tandem mass spectrometry (HPLC-MS/MS) can be used to simultaneously quantify up to 121 pesticide residues, including carbamates and organophosphorus compounds. Post-harvest cleaning has been reported to reduce pesticide residues by approximately 40–60% [46]. Mercury contamination in rice and rice-derived products has been assessed using atomic absorption spectrometry combined with toxicological models. The results suggest that total mercury content is highest in rice cake and lowest in rice and rice flour, with all values remaining below the safety threshold (THQ < 1). These findings indicate that current consumption levels do not pose a significant health risk; however, the potential for long-term bioaccumulation among individuals with high rice consumption warrants continued attention [47].

In contrast to chemical contamination, mycotoxin contamination is regarded as a more concealed and long-term threat to rice safety, due to the thermal stability of mycotoxins and their potential carcinogenic properties. Rice is considered particularly susceptible to mycotoxin contamination due to its high starch content, with aflatoxins, fumonisins, ochratoxins and deoxynivalenol among the most frequently detected mycotoxins [48]. Additionally, coloured rice is characterised by a high content of anthocyanins and other bioactive compounds, and its processing and storage conditions may aggravate the risk of mycotoxin contamination. A weak correlation between anthocyanin content and aflatoxin B1 levels has been reported; however, the underlying mechanism and causal relationship remain unclear and require further examination [49]. Fungal toxins have been shown to exhibit higher sensitivity to specific spectral bands in hyperspectral imaging (HSI). When HSI is combined with machine-learning algorithms, the accuracy of fungal toxin detection in rice has improved [50].

A magnetic nano-adsorbent composed of natural carboxymethyl cellulose, polyaniline nanotubes and graphene oxide has been reported to efficiently adsorb aflatoxins B1 and B2 in rice through π–π interactions, hydrogen bonding and hydrophobic interactions. Its adsorption performance has been shown to be superior to that of conventional solid-phase extraction techniques [51]. With respect to control strategies for mycotoxin contamination, several decontamination approaches have been explored, including microwave treatment, pulsed light, ozone, cold plasma and irradiation, as well as the application of plant-derived antifungal metabolites and microbial biocontrol methods [52]. Additionally, the application of carbon monoxide (CO) at a concentration of 50% has been reported to completely inhibit the growth of Aspergillus flavus and Fusarium proliferans in rice, while exposure to 70% CO has been shown to prevent fungal survival in milled rice. The inhibitory effect on toxin production was reported to be stronger than that achieved by bacterial control; however, the potential impact of CO treatment on the sensory properties of rice requires further verification [53].

## 4. Effects of Ecological Factors and Management on Rice Quality and Improvement Measures

**Figure 4:**
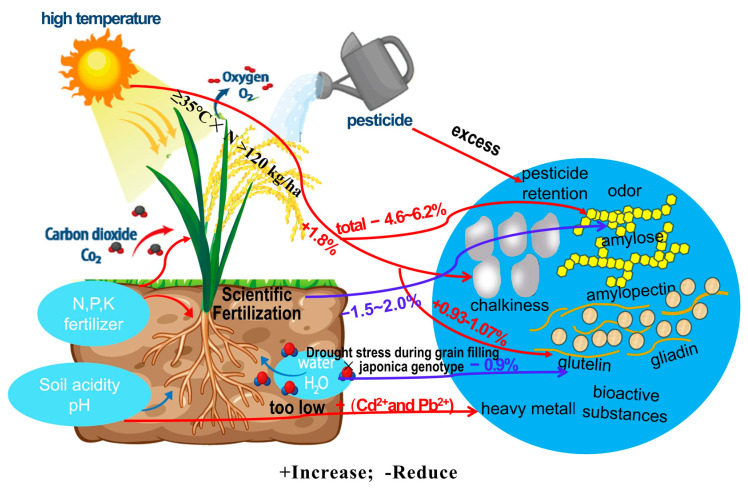
Influence of environment and management factors on rice quality.

Rice quality is regulated by multiple interacting factors that are expressed through environmental and management conditions. The principal ecological and agronomic factors influencing rice quality include climate, fertilisation practices, soil environment, water management and pesticide application (Figure 4).

### 4.1. Effects of Climate and Environment on Rice Quality

The dynamic combination of climatic factors during the grain-filling stage has a direct impact on rice quality, primarily through the regulation of starch synthase activity and its effect on chalkiness formation. When the daily average temperature exceeds 30 °C during grain filling, high temperatures inhibit normal starch synthesis and accumulation, resulting in an irregular arrangement of starch particles that aggravate chalkiness. Simultaneously, high temperatures may induce an abnormal increase in protein content, negatively impacting rice eating quality. However, low temperatures (<18 °C) during the grain-filling stage significantly reduce photosynthetic activity and carbon metabolism, slow dry matter accumulation, increase chalkiness and decrease the head rice yield.

High temperatures during rice grain filling disrupt endosperm starch biosynthesis, reducing rice quality. The interaction between starch biosynthesis enzymes *OsGBSSI* and *OsPPDKB* with the *OsHsp40-1* gene enhances enzyme stability and activity, thereby improving rice quality and yield under moderately high temperatures [54]. Under climate scenarios RCP4.5 and RCP8.5, global warming has significantly affected rice development across various growth stages, contributing to the deterioration of rice quality. High temperatures have been reported to substantially increase chalkiness by approximately 1.6–3.1%, elevate protein content by approximately 0.93–1.07%, and raise gelatinisation temperature and retrogradation, while reducing total starch content by approximately 4.6–6.2%, collectively diminishing rice quality [55]. Elevated atmospheric CO_2_ can significantly improve the activity of soluble starch synthase and granule-bound starch synthase (GBSS), increase starch granule chain length and volume and modify starch morphology and structure, thereby altering starch thermal properties and ultimately improving cohesiveness and taste [56]. Additionally, the high-temperature-responsive UDP-glycosyltransferase gene *OsUT72F1* was isolated from rice; its activity induced the up-regulation of multiple metabolic pathways, including phenylpropane biosynthesis, zeatin biosynthesis and flavonoid biosynthesis, thereby improving the nutritional characteristics of rice [57].

### 4.2. Effect of Fertilisation on Rice Quality

During field management, the content and distribution of protein and amylose in rice were significantly influenced by the judicious application of nitrogen and potassium fertilisers, whereas eating quality was adversely affected by excessive or insufficient application [58]. Nitrogen top-dressing at the heading stage was required to adhere to the principles of ‘timeliness and appropriateness’ and was recommended to be applied 5–7 d before heading. Potassium sulfate as a basal fertiliser was required to be applied at 30–35% of the total base fertiliser amount, corresponding to 37.5–45.0 kg/hm^2^. Rice protein content was maintained at 6.5–7.2%, chalkiness was reduced by 2.8–3.2% and 1000-grain weight was increased by 1.2–1.5 g through this practice. Additionally, supplementary potassium fertiliser was applied at the grain-filling stage, 5–7 d after heading, using potassium sulfate at a rate of 30.0–37.5 kg/hm^2^, delivered through ‘spraying + foliar spraying’. Through this approach, rice amylose content was reduced by 1.5–2.0%, grain length was increased by 0.2–0.3 mm, grain width was increased by 0.1–0.2 mm and head rice rate improved by 2.8–3.2%.

Reduced nitrogen and phosphorus fertilisation diminished the activities of ADP-glucose pyrophosphorylase and GBSS in japonica rice, thereby inhibiting amylose accumulation and improving eating quality [59]. The application of controlled-release urea in place of conventional urea reduced chalkiness, protein content and starch crystallinity, thereby enhancing both the appearance and eating quality of rice [60].

### 4.3. Effects of Soil Environment on Rice Quality

Soil, as the basic substrate for rice growth, contains numerous ecological factors that critically influence rice quality, including soil texture, nutrient content (e.g., nitrogen, phosphorus, potassium and other elements), pH and topsoil depth. A soil environment with a deep plough layer, good aeration and high water- and nutrient-retention capacity provides a crucial foundation for achieving high rice yield and quality. Approximately 80–90% of rice mineral nutrition is derived from the soil, and its abundance, deficiency and balance directly affect the processing quality, appearance and nutritional composition of rice by regulating plant physiological metabolism and biosynthetic pathways. Soil pH is a key regulator of rice quality, with the optimal range being 5.5–6.5. Under acidic conditions, the proportion of exchangeable heavy metals, such as cadmium (Cd^2+^) and lead (Pb^2+^), was increased two- to threefold, thereby significantly enhancing their translocation to rice grains and posing serious risks to food safety.

Furthermore, in moderate saline soils, rice eating quality is primarily affected through increased chalkiness, reduced amylose content and increased protein content. This is because chalkiness-related gene *Chalk5* is up-regulated, the amylose-related gene Wx is down-regulated and protein-related genes *OsAAP6* and *OsGluA2* are up-regulated under salinity during the growth of hairy rice [61]. Additionally, *OsDSR3*, a member of the *DUF966* gene family, positively regulates tolerance to alkali stress in rice [62]. At the germination stage, six differentially expressed genes [63] and eight *Os* genes [64] were identified as contributors to improved salt and alkali tolerance.

### 4.4. Effect of Water Management on Rice Quality

Rice growth is often affected by fluctuations in soil moisture, and improving yield and quality under water-saving conditions is a key objective in rice breeding. Water management practices significantly impact rice quality. First, shallow-water irrigation at the tillering stage was conducted by maintaining a 1–2 cm water layer for 3–4 d, followed by field drying for 2–3 d until cracks 1–2 mm deep were observed. Second, wet irrigation was applied at the booting stage. During early booting, the field water level was reduced to 2–3 cm. When young panicles reached 0.5–1.0 cm in length, wet irrigation was employed to maintain soil moisture without causing waterlogging, with soil water content controlled at 85–90% of field capacity. Third, alternate wetting and drying during the grain-filling period was initiated with a 3–4 cm shallow water layer maintained for 2–3 d to promote the translocation of photosynthetic products to the grains. This was followed by cycles of keeping the field wet for 3 d and moderately drying it for 2 d. These water management practices were observed to increase the effective panicle number by 15–20% and reduce protein content by 0.8–1.2%, thereby significantly improving rice eating quality.

Genetic improvement can further enhance yield and eating quality under fluctuating soil moisture. For example, the *G3-3* gene has been shown to reduce protein and amylose content but increase the broken rice rate, a finding that requires further verification [65]. The results showed that drought at specific developmental stages significantly reduced grain size, bulk density and the contents of starch, amylose, amylopectin and soluble protein. Additionally, drought was found to alter the accumulation of trace elements (Cu, Fe, Mn, Na and Zn) and major elements (P, K, Ca and Mg) in rice [66]. Following drought stress, the percentages of chalkiness, amylose (16.57–20.999%) and protein (7.99–12.09%) were increased, whereas appearance quality and eating quality were decreased [67]. Under aerobic cultivation, drought-tolerant genotypes (*Giza-179*, *Hybrid-1*, *Giza-178* and *Line-9399*) were observed to yield more and maintain higher quality than drought-sensitive genotypes, while reducing water consumption [68].

Water quality is also critical to rice quality, particularly in terms of salt and mineral element content. Optimal mineral concentrations were observed to positively influence rice quality; for example, trace elements such as iron and manganese were found to enhance enzyme activity, thereby supporting starch and protein synthesis and improving both nutritional and appearance quality. However, excessive concentrations can be toxic and negatively affect rice quality. For example, when irrigation water containing Fe^2+^ ≥ 5 mg/L or Mn^2+^ ≥ 3 mg/L was applied, heavy metal accumulation in rice was increased, potentially compromising safety and quality.

### 4.5. Effect of Pesticides on Rice Quality

During rice growth and development, crops are often exposed to pest and disease pressure, particularly during the middle and late growth stages. Therefore, pesticide application has become a routine practice in agriculture. However, concerns have been raised regarding the potential adverse effects on rice quality and the presence of pesticide residues. Gas chromatography–mass spectrometry was used to simultaneously detect 15 pesticides in rice samples. The results showed that the detection rates of these pesticides ranged from 0% to 12.9% in rice and from 0% to 1.4% in brown rice, with all detected residues remaining below the maximum residue limits specified by Chinese regulatory standards. Among the detected compounds, chlorpyrifos exhibited the highest detection frequency and concentration [69]. Pymetrozine, a pyridylazo methyl insecticide, has been reported to be effective in controlling planthoppers. However, variations in rice appearance, taste and aroma have been observed under different pesticide treatment regimens [70]. It has been reported that pests can be effectively controlled through the application of 25% pymetrozine and chlorpyrifos suspension concentrate, administered twice at 10 d intervals within a dosage range of 375–562.5 g a.i./ha. Rice was reported to be safely harvestable 15 d after the final application of pymetrozine and chlorpyrifos [71].

In summary, rice quality is significantly influenced by the growth environment and field management practices. Through genetic improvement via breeding and optimised field management strategies, the adverse effects of unfavourable environmental conditions on rice quality can be alleviated (Table 3).

## 5. Improvement of Rice Quality Through Genetic Improvement Technology

With advances in molecular breeding, the eating quality of hybrid rice has been substantially improved in China over the past 40 years. This improvement has largely been attributed to the targeted utilisation of dominant alleles influencing grain quality traits [72]. Molecular marker technologies, multi-omics approaches and gene-editing techniques have been widely applied in rice molecular breeding research (Table 4).

### 5.1. Identification of Rice Appearance Quality-Related Genes

Improving the appearance quality of rice is critical for market acceptance. Grain shape is a typical quantitative trait controlled by multiple genes, which may exhibit epistatic or additive effects [73]. The genes *qGL4-2*, *qGWh5* and *qGWh10* have been identified as key contributors to grain shape [74]. The formation of chalkiness is genetically regulated. A total of 34 quantitative trait loci (QTLs) associated with grain chalkiness were identified through a genome-wide association study, including *14 PGWC QTLs* and *20 DEC QTLs*, with *Os10g36170*, *Os10g36260*, *Os10g36340* and *Os10g366100* identified as candidate genes [75]. High expression of genes *Nip (Wxb/SSII-2)* and *Nip (Wxb/ss2-2)* has been associated with increased rice particle transparency [76]. Genome-wide association analysis identified two candidate genes, encoding *MAPKK6* and *OPAQUE3*, as significantly associated with rice pericarp colour [77]. Additionally, four functional candidate genes (*Os01g66110*, *Os01g66140*, *Os07g44910* and *Os02g14120*) were found to be closely related to aroma, head rice rate and chalkiness and may be employed in rice molecular breeding to improve appearance quality traits [78]. The genes *Os05g06920*, *Os05g06970* and *Os11g28104* were observed to increase grain length and reduce grain width, whereas *Os050g06970* and *Os11g28104* significantly increased the chalkiness percentage and grain whiteness [79].

### 5.2. Identification of Rice Eating Quality-Related Genes

Amylose-to-amylopectin content and protein content are directly associated with rice eating quality. The expression of starch synthesis-related genes in the endosperm predominantly controls these traits. Synergistic down-regulation of *SSII-2* and *SSII-3* expression has been shown to reduce amylose content, thereby improving rice eating quality [80]. Amylopectin content in rice is primarily regulated by the *Waxy* (*Wx*) gene; for example, waxy rice carries *Wxmp*, *Wxmq* or *Wxb-5c* alleles [81]. Glutenin-related genes in rice are complex and highly sensitive to environmental conditions. The deletion of genes encoding glutenin through *CRISPR/Cas9* was observed to reduce glutenin content and improve eating quality [82]. Molecular genetics, plant biotechnology and functional genomics studies have revealed that the *OsMADS1^Olr^* mutant allele contributes to poor rice quality, manifesting as high endosperm chalkiness, abnormal starch granule morphology and loose granule arrangement, and high protein content. Therefore, *OsMADS1* is recognised as a regulatory gene associated with starch and protein metabolism, influencing rice eating quality [83]. Resistant starch content in traditional rice is approximately 2%; however, the loss of *SSIIa*, *SSIVb* or *ISA2* was observed to increase resistant starch content to over 14%. Quadruple mutants comprising *sbeI*, *sbeIIb*, *ssIIIa* and *ssIIIb* and *sbeI*, *ssIVb*, *ssIIIa* and *ssIIIb* were reported to further increase resistant starch content to over 18%, accompanied by a significant decrease in the eating quality of cooked rice [84].

### 5.3. Identification of Active Ingredient Related Genes in Rice

Genes regulating polyphenol content in rice seeds have not been fully characterised; however, in the seed coat of *Arabidopsis thaliana*, *OsCHS*, *OsCHI*, *OsF3H*, *OsF30H*, *OsDFR* and *OsANS* have been shown to influence pigmentation, converting yellow to purple [85]. Flavonoids, a subclass of polyphenols, are similarly regulated. Expression analysis of flavonoid biosynthesis genes in rice has shown that *OsSPL17* regulates *CHI* expression, thereby increasing the accumulation of flavanones (eriodictvol and naringenin) and flavonoids (apigenin and luteolin), while also contributing to overall rice growth and development [86]. Under heat stress, the glycosyltransferase gene *OsDUGT1* has been identified as a heat-responsive gene in rice, catalysing the glycosylation of flavonoid compounds. Correspondingly, high expression of *OsDUGT1* significantly increased endogenous flavonoid accumulation [87]. *OsCOP1* is involved in embryonic development and flavonoid biosynthesis in rice grains; its overexpression in the *yel-hc* mutant restored peel colour, while flavonoid accumulation remained abnormal in the embryo [88]. Co-expression of *ZlRc* and *ZlRd* increased flavonoid content and antioxidant activity without affecting agronomic traits or yields, while simultaneously inhibiting the activities of α-glucosidase, α-amylase, pancreatic lipase and tyrosinase [89].

The synthesis of GABA in rice is catalysed by glutamic acid decarboxylase (GAD). The genes *OsGAD1* and *OsGAD3* can truncate the calmodulin-binding domain of GAD, enhancing enzyme activity and promoting GABA accumulation [90]. During germination, the GABA content of brown rice is significantly increased, which is attributed to the involvement of GAD cDNA genotypes *OsGAD1*, *OsGAD2* and *OsGAD3* in GABA biosynthesis, with *OsGAD3* exhibiting the highest up-regulation [91]. In rice varieties with high GABA content, GABA accumulation is associated with the up-regulation of *OsGAD* transcription, up-regulation of polyamine pathway intermediates and down-regulation of GABA catabolism transcription genes [92]. Genetic breeding approaches have also been employed to increase the concentration of selected vitamins in rice, such as vitamin A, by targeting key biosynthetic enzymes [93].

### 5.4. Artificial Inteligence (AI) in the Rice Quality

During the growth process of rice, it is highly susceptible to diseases such as leaf blight, leaf blight, and brown spot, which can significantly reduce yield and affect rice quality. AI, particularly computer vision and machine learning, has developed a convolutional neural network (CNN) based model for rice leaf disease detection and classification. In order to improve the accuracy of disease classification, CNN enhances spatial and channel attention mechanisms, allowing it to focus on the most discriminative image regions, achieving high accuracy and robust performance indicators. The attention mechanism significantly improves the accuracy of identifying subtle disease patterns [94].

Traditional rice quality assessment methods are gradually integrating artificial intelligence. Currently, AI applications, including machine learning, deep learning, spectroscopy, thermal imaging, and hyperspectral imaging, are used to evaluate and classify the quality of rice at various processing stages. Convolutional neural networks (CNN), YOLO architecture, and Mask R-CNN models have been integrated into industrial rice milling systems, and the classification accuracy for grading and variety identification is usually ≥90%. Near-infrared artificial star neural network models for physical and chemical properties (such as moisture/protein) often report strong fitting (R^2^ ≈ 0.90–0.99) [95].

## 6. Discussion

### 6.1. Synergistic and Antagonistic Effects of Environmental Factors: A Complex System

This review systematically examines the effects of factors such as temperature, light, water, and soil on rice quality, with the key being their synergistic and antagonistic effects. For example, the synergy of temperature and light: strong light during the grain filling period combined with a suitable temperature difference between day and night can maximise the high-quality synthesis of starch and protein and enhance the taste value. But if accompanied by high temperatures, it may exacerbate chalkiness and counteract the positive effects of light exposure. The interaction of water and fertiliser management: excessive application of nitrogen fertiliser under water stress conditions can significantly deteriorate the appearance quality of rice, and its negative impact is far greater than any single factor. The coupling between soil and climate: whether selenium rich soil can produce stable levels of selenium rich rice depends greatly on local precipitation patterns and irrigation water quality. Future research needs to use more multi factor orthogonal experiments and ecosystem simulations to reveal the networked mechanisms of environmental impacts.

### 6.2. Threshold and Nonlinear Response of Key Environmental Stress

The impact of the environment on quality often exhibits threshold effects and nonlinear responses. For example, when the temperature exceeds 35 ℃ during the grouting period and reaches a critical value, the chalkiness rate and whole grain rate will sharply decrease; the cadmium content in soil has a weak impact on quality when it is below the safety standard, but once it exceeds the standard, its accumulation rate and quality deterioration will suddenly intensify. Determining the safety thresholds and sensitive window periods for these key environmental parameters (such as 10–20 days after pollination, which are most sensitive to high temperatures) is of crucial practical significance for developing precise agronomic control measures. However, these thresholds face challenges in establishing universal models due to significant differences in variety and region.

### 6.3. Genotype (G) × Environment (E) Interaction: The Core of Quality Stability

Rice quality is a typical quantitative trait that is influenced by the strong interaction between genotype and environment (G × E). The same variety may exhibit vastly different quality characteristics in different environments. Variety adaptability has specialisation: certain high-quality varieties (such as Koshiguang) can only exhibit their best quality in specific ecological areas (such as Niigata in Japan and Jilin in China), which is a perfect match between specific genotypes and optimal environmental conditions. Breeding strategies need to be adjusted; modern quality breeding must be carried out under the framework of multi environment testing (MET), screening genotypes that perform robustly in different environments, or cultivating specialised varieties for specific advantageous ecological areas. Frontiers in molecular mechanism research: through association analysis, QTL mapping, and transcriptomics, the discovery of major QTLs and molecular markers that control quality stability is crucial for achieving molecular design breeding and cultivating “environmentally intelligent” high-quality varieties.

### 6.4. Contradictions, Gaps, and Uncertainties in Current Research

Contradictory point: for example, regarding the effect of nitrogen fertiliser on taste value, some studies suggest that increasing nitrogen reduces taste, while others believe that it can improve it in specific contexts. This may be due to differences in variety, nitrogen fertiliser form (ammonium/nitrate), application period, and different ratios with other elements. More refined experimental design is needed to distinguish.

Research gap: long term impacts of climate change; insufficient research on the long-term trends of elevated CO_2_ concentrations and increased frequency of extreme weather events (heat waves, floods) on the nutritional quality of rice (such as protein and trace element content). Attention to new pollutants: there is more attention paid to traditional heavy metals, but research on the accumulation patterns of emerging pollutants such as microplastics, antibiotics, and persistent organic pollutants in rice and their impact on quality is just beginning. The impact of postpartum environment: the review mainly focuses on the pre-production field environment, but the effects of postpartum drying and storage (temperature and humidity) environment on rice cracking, yellowing, and ageing rate are also significant, and systematic research in this area is relatively weak.

## 7. Conclusions

Artificial intelligence (AI) technology enables the integration of multidimensional data, including genetic, environmental and management factors, to construct a ‘digital twin’ model. This model can simulate the performance of various hybrid combinations across diverse environments and provide breeders with optimal strategies for parental and progeny selection. Complex quantitative traits are controlled by multiple genes and strongly influenced by environmental conditions, such as drought tolerance and nitrogen use efficiency. Multi-location validation of OsGAD3 overexpression effects on GABA content under combined drought + heat stress (≥35 °C), given that 87% of current studies test single stressors. High temperature weather can lead to drought and increase the difficulty of field water management, resulting in 1.6–3.1% increase in rice chalkiness, 0.93–1.07% increase in protein content, 4.6–6.2% decrease in total starch content, and ultimately a decline in the appearance and taste quality of rice.

Field trials remain the gold standard for validating and refining AI prediction models. Continuous monitoring throughout the growth period and standardised management practices are essential to ensure that observed differences are primarily attributable to genotypes. Environmental variability and interannual climate fluctuations may obscure genetic effects; therefore, experiments should be conducted across multiple locations and seasons to enable rigorous evaluation of genotype–environment interaction effects; for example, the impact of OsGAD3 overexpression on GABA content under drought stress must be verified under diverse conditions. Genes may perform optimally in one region but exhibit limited or adverse effects in another, highlighting the need for further in-depth research on interaction mechanisms and the application of AI modelling for adaptive breeding. Socioeconomic barriers to technology adoption must be addressed (e.g., smartphone-based field management tools require low-bandwidth design for smallholder farmers). Under suitable climatic conditions, good water management measures increase the effective number of rice panicles by 15–20% and reduce protein content by 0.8–1.2%, thereby significantly improving rice yield and taste quality. Genetic improvement can alleviate the changes in rice quality caused by water fluctuations in unstable soil moisture.

The development of a rice field management programme using remote sensing technology integrates the internet of things, big data, AI and agronomic knowledge. This approach enables a transition from ‘extensive management’ to ‘precision prescription’, transforming conventional uniform field management into a precise, closed-loop strategy encompassing ‘monitoring, diagnosis, prescription and execution’. Such integration enables the visualisation, quantification, early warning and informed decision-making of the entire rice growth process. The ultimate objective is the sustainable advancement of rice production through ‘enhanced yield, reduced costs, improved quality and environmental protection’, representing the inevitable trajectory of smart agriculture.

## Figures and Tables

**Table 1:** Quality indicators of first grade rice in China.

Variety		Indica Rice	Japonica Rice	Indica Glutinous Rice	Japonica Glutinous Rice
Broken rice, total (%)		≤15.0	≤10.0	≤15.0	≤10.0
	Small broken rice (%)	≤1.0	≤1.0	≤2.0	≤1.5
Processing accuracy		Fine milling	Fine milling	Fine milling	Fine milling
Unsound kernel (%)		≤3.0	≤3.0	≤4.0	≤4.0
Moisture content (%)		≤14.5	≤15.5	≤14.5	≤15.5
	Impurities, total (%)	≤0.25	≤0.25	≤0.25	≤0.25
	Inorganic impurities (%)	≤0.2	≤0.2	≤0.2	≤0.2
Yellow-coloured rice (%)		≤1.0	≤1.0	≤1.0	≤1.0
Mixing rate (%)		≤5.0	≤5.0	≤5.0	≤5.0

**Table 2:** Pesticides commonly used in rice cultivation.

Category	Name	Primary Purpose
Fungicides	Tricyclic azole	Used for the prevention of rice blast, including leaf blast and neck blast.
	Rice distemper	Used for both the prevention and treatment of rice blast disease, with preventive and curative effects.
	Jinggangmycin	Applied for the prevention and control of sheath blight and rice blast.
	Azoxystrobin/Pyrazoxystrobin	Broad-spectrum fungicides used to control rice blast, sheath blight and related fungal diseases.
	Hexazolol and benzofenapyr	Used for the prevention and treatment of rice sheath blight and rice blast.
	Chunlei mycin	Applied for the control of rice blast and bacterial stripe disease.
Insecticides	Imidacloprid and Thiamethoxam	Used to control rice planthoppers and thrips; classified as neonicotinoids.
	Chlorfenapyr	Applied for the control of rice leaf rollers and stem borers (Lepidoptera pests).
	Abamectin	Used for the prevention and control of rice stem borers and leaf rollers.
	Chlorpyrifos	Broad-spectrum insecticide used to control borers and rice planthoppers; use is restricted in some countries.
	Fipronil	Applied for the control of rice planthoppers and leafhoppers.
	Imidacloprid	Used to control rice leaf rollers and stem borers.
	Ethyl fungicide	Applied for the prevention and control of borers and thrips.
Herbicides	Dichloraz and Propiconazole	Pre-emergence herbicides used to control barnyard grass and annual weeds.
	Bensulfuron methyl and pyrimethanil	Used to control broad-leaved weeds and sedges.
	Pentafluorosulfonamide	Broad-spectrum herbicide applied to control barnyard grass and broad-leaved weeds.
	Quinclorac	Specifically used to control barnyard grass.
	Cyanofloxacin	Applied to prevent and control grass-family weeds, including barnyard grass and millet species.
	Oxalidomide	Used to prevent and control herbicide-resistant barnyard grass and horseweed.
Growth regulators	Paclobutrazol	Applied to suppress excessive vegetative growth and promote tillering.
	Gibberellin	Used to promote heading and flowering.

In China, the maximum residue limit of pesticides in food is implemented in accordance with GB 2763-2021 “National Food Safety Standard—Maximum Residue Limits of Pesticides in Food”.

**Table 3:** Impact of environmental factors on rice quality and corresponding improvement measures.

Environmental Factors	Changes in Rice Quality	Improvement Measures
Climate	High temperature was associated with increased chalkiness and protein content, and with declines in appearance and eating quality.	Introduction of the *OsGBSSI*, *OsPPDKB* and *OsHsp40-1* gene enhances enzyme stability and activity, has been reported to reduce chalkiness by approximately 2.8–3.2% under high-temperature conditions (n = 3 studies).
Fertilisation	Excessive or insufficient nitrogen fertiliser application led to unfavourable changes in starch composition, protein content and chalkiness, thereby reducing rice quality.	Nitrogen fertiliser application at the heading stage (5–7 d before heading) at 120–150 kg/ha has been reported to achieve an optimal protein content of 6.5–7.2%. Seven studies showing improved eating quality vs. five showing deterioration by analysing methodological differences (drought timing: booting vs. grain filling; genotype backgrounds.
Soil	Saline–alkaline soils were associated with adverse effects on chalkiness, starch and protein contents, resulting in reduced rice quality.	A soil pH of 5.5–6.5 has been reported to reduce the accumulation of heavy metals, such as Cd^2+^ and Pb^2+^, by approximately 50%. Downregulation of the *chalk5* gene has been associated with reduced chalkiness; downregulation of *Wx* with reduced amylose content and downregulation of *OsAAP6* and *OsGluA2* with reduced protein content.
Moisture	Drought stress was associated with adverse effects on bulk density, chalkiness, starch, protein and trace element contents, leading to reduced quality.	During the tillering stage, maintaining a water layer of 1–2 cm for 3–4 d has been reported to be beneficial. During the booting stage, the water level has been reduced to 2–3 cm. During the grain-filling stage, maintaining a 3–4 cm water layer for 2–3 d followed by moderate field drying for 2 d has been reported to reduce protein content by 0.8–1.2%. Introduction of the *G3-3* gene has been reported to reduce protein and amylose content. The application of Moringa oleifera leaf extract has been reported to alleviate drought-related decline in rice quality.
Pesticide	Application of the insecticide pymetrozine was associated with changes in rice appearance, taste and aroma.	Spraying a 25% pymetrozine and chlorpyrifos suspension twice at 10 d intervals, at a dose of 375–562.5 g a.i/ha, has been reported. Rice was harvested 15 d after the final application to ensure that pesticide residues remained within a safe range.

**Table 4:** Identification of rice quality-related genes.

Quality Type (Study Counts)	Index	Related Gene and Effect Size
Appearance (*n* = 7)	Shape	The *qGL4-2*, *qGWh5* and *qGWh10* genes control rice grain shape; the *Os05g06920*, *Os05g06970* and *Os11g28104* genes increase grain length while decreasing grain width.
	Chalkiness	The *Os10g36170*, *Os10g36260*, *Os10g36340* and *Os10g36610* genes increase chalkiness; whereas *Ss050g06970* and *S11g28104* reduce chalkiness.
	Transparency	High expression of *Nip (Wxb/SSII-2)* and *Nip (Wxb/ss2-2)* genes improves the transparency of rice grains.
	Colour	The *MAPKK6* and *OPAQUE3* genes are associated with rice colour, with *MAPKK6* linked to red and *OPAQUE3* to white grains.
Taste (*n* = 5)	Starch	Low expression of *SSII-2* and *SSII-3* reduces amylose content. High expression of *Wxmp*, *Wxmq* and *Wxb-5c* increases amylopectin content. The absence of *SSIIa*, *SSIVb* or *ISA2* increases resistant starch content to over 14%, while quadruple mutants (*sbeI*, *sbeIIb*, *ssIIIa* and *ssIIIb*, and *sbeI*, *ssIVb*, *ssIIIa* and *ssIIIb*) further increase resistant starch content to over 18%.
	Protein	Knockout of *CRISPR/Cas9* gene reduces the content of valley proteins, whereas *OsMADS1* regulates protein content in rice.
Nutrition (*n* = 5)	Polyphenols and flavonoids	*OsCHS*, *OsCHI*, *OsF3H*, *OsF30H*, *OsDFR* and *OsANS* increase polyphenol content and modify seed colour. *OsSPL17* upregulates flavones (eriodecvol, naringenin, apigenin and luteolin), while *OsJRL* knockout increases flavonoid content. *OsCOP1* promotes flavonoid accumulation, accumulation of *yel-hc* enhances flavonoid content in the embryo and synergistic high expression of *ZlRc* and *ZlRd* increases both flavonoid content and antioxidant activity.
	γ-aminobutyric acid	Expression of the *OsGAD1*, *OsGAD*, and *OsGAD3* genes increased GABA content, with *OsGAD3* exhibiting the most significant up-regulation.

## Data Availability

No new data were created or analysed in this study.
